# Development of educational technology on sexually transmitted infections for adolescent students

**DOI:** 10.1590/0034-7167-2025-0024

**Published:** 2026-03-16

**Authors:** Mayra Loreanne Nascimento Corrêa, Maria Eduarda dos Santos Alves, Pablo Palmerim Santana, Vinícius dos Santos Maciel, Angélica Gonçalves Silva Belasco, Tatiana do Socorro dos Santos Calandrini, Camila Rodrigues Barbosa Nemer, Nely Dayse Santos da Mata

**Affiliations:** I Universidade Federal do Amapá. Macapá, Amapá, Brazil; II Universidade Federal de São Paulo. São Paulo, São Paulo, Brazil

**Keywords:** Adolescent, Sexually Transmitted Diseases, Health Literacy, Educational Technology, Health Education., Adolescente, Enfermedades de Transmisión Sexual, Alfabetización en Salud Tecnología Educacional, Educación en Salud.

## Abstract

**Objectives:**

to describe the creation of an educational booklet for adolescent students on the main sexually transmitted infections.

**Methods:**

this methodological and quantitative study was conducted in four stages: 1. Assessment of adolescent students’ health literacy level based on the Short Test of Functional Health Literacy in Adults; 2. Literature survey; 3. Content analysis and text elaboration; 4. Booklet development. The research followed the Strengthening the Reporting of Observational Studies in Epidemiology checklist.

**Results:**

only 8.1% of adolescents have adequate health literacy. The booklet was divided into: What are sexually transmitted infections?; Learn about the main sexually transmitted infections; I discovered I have a sexually transmitted infection, what should I do?; How can I prevent a new infection?

**Conclusions:**

the booklet on sexually transmitted infections shows potential to contribute to health education practices, facilitating adolescents’ understanding of the topic.

## RESUMEN

**Objetivos:**

describir el desarrollo de una cartilla educativa para escolares adolescentes sobre las principales infecciones de transmisión sexual.

**Métodos:**

este estudio metodológico, con un enfoque cuantitativo, se llevó a cabo en cuatro etapas: 1. Evaluación del nivel de alfabetización en salud de estudiantes adolescentes mediante el Short Test of Functional Health Literacy in Adults; 2. Revisión bibliográfica; 3. Análisis de contenido y elaboración textual; 4. Elaboración del folleto. La investigación siguió el checklist STrengthening the Reporting of OBservational studies in Epidemiology.

**Resultados:**

solo el 8,1% de los adolescentes tienen un nivel adecuado de alfabetización en salud. El folleto se dividió en: ¿Qué son las infecciones de transmisión sexual?; Conozca las principales infecciones de transmisión sexual; Descubrí que tengo una infección de transmisión sexual, ¿qué debo hacer?; ¿Cómo puedo prevenir una nueva infección?

**Conclusiones:**

la cartilla sobre infecciones de transmisión sexual muestra potencial para contribuir a las prácticas de educación para la salud y facilitar la comprensión de los adolescentes sobre el tema.

## INTRODUCTION

The Ministry of Health considers the definition used by the World Health Organization, which characterizes adolescence as the period from 10 to 19 years old, and understands youth as the population aged 15 to 24 years old^(1)^. This period is marked by several intense biological, psychological, and social changes, and it marks the beginning of sexual life for many young people. Epidemiological studies indicate that adolescents represent a vulnerable group at risk for contracting sexually transmitted infections (STIs) due to several factors, such as early sexual initiation, multiple sexual partners, and irregular condom use^(2,3)^.

Associated with this, many adolescents lack an adequate understanding of the risks and ways to prevent STIs, which directly contributes to the spread of these infections and increases the vulnerability of this group. This lack of understanding is compounded by stigma and taboo that still surround the topic of sexuality in society. Often adolescents lack a safe environment to discuss their doubts and concerns, whether with family, friends, or even at school. This communication barrier leads many young people to seek information from unreliable sources or, in some cases, not even seek information at all, adopting practices without proper knowledge^(4)^.

This lack of understanding can also be understood as a lack of health literacy (HL), which refers to individuals’ ability to understand, assess, and use health information to make decisions about their health. In the context of STIs, this can include knowledge about how they are transmitted, prevention methods, and recognition of signs and symptoms. When individuals have access to clear information tailored to their circumstances, they feel more empowered and able to adopt preventive measures, seek professional help when necessary, and demystify stigmas that can delay diagnosis and treatment^(5)^.

Thus, it is understood that the lack of access to health information about STIs is a key issue that has contributed to the high prevalence of these infections in adolescents. Therefore, an individual with adequate HL is more likely to have better health conditions than one with inadequate HL, who has more difficulty understanding guidance regarding preventive measures and their own health^(5)^.

Therefore, the need to develop educational strategies that promote understanding and awareness of STIs among adolescents is emphasized. Educational technologies, such as brochures and booklets, when aimed at adolescents, are resources that enhance learning in a playful manner, promoting a change in behavior that strengthens the protection of young people’s sexual health and contributes to reducing STI infection rates in this age group^(6,7)^.

## OBJECTIVES

To describe the development of an educational booklet for adolescent students on the main STIs.

## METHODS

### Ethical aspects

The research followed the standards of Resolution 466/12 of the Brazilian National Health Council, which establishes guidelines and regulatory standards regarding the ethical aspects of research, and was submitted to and approved by a Research Ethics Committee. All research participants signed the Informed Consent Form and the Informed Assent Form, of which two copies (originals) were signed. One copy remained with participants, and the other with researchers.

### Study design, period and location

This is a methodological and quantitative study developed in four stages^(8,9)^: 1. Assessment of adolescent students’ HL level based on the Short Test of Functional Health Literacy in Adults (S-TOFHLA) instrument; 2. Literature survey; 3. Content analysis and text elaboration; 4. Booklet development. The research followed the STrengthening the Reporting of OBservational studies in Epidemiology checklist.

This study was carried out between August 2022 and May 2023 in 11 public schools located in Macapá, capital of the state of Amapá, divided into state and municipal schools.

### Population or sample; inclusion and exclusion criteria

The study involved 558 adolescent students from 11 public schools in the municipality of Macapá. Participants were selected by convenience during the data collection period, through personal invitations to participate in the study by the researchers in classrooms, who provided detailed and clear explanations of the research and its objectives.

In each of the schools, there were approximately 350 students within the established age range, resulting in a total of approximately 3,850 adolescents. The schools were selected based on recommendations from the Department of Education. To ensure the project’s viability, two schools were selected per geographic zone (north, south, east, and west), resulting in six state schools and five municipal schools.

Sample size calculation was not performed in advance, given the exploratory nature and the feasibility of the research in partnership with the schools. However, considering an estimated population of 3,850 adolescents enrolled in the 11 participating schools, the final sample of 558 students accounted for approximately 14.5% of the eligible population, providing good representation for local assessment.

Adolescents between 15 and 18 years old, enrolled in a state or municipal public school in the state of Amapá, and regularly attending classes were included. Adolescents with self-reported cognitive impairment, reported by the school, or who changed schools during the study period, were excluded.

### Study protocol

The first stage is integrated into the research project entitled “*Letramento em saúde entre escolares de 15 a 18 anos na saúde sexual e reprodutiva da rede pública no estado do Amapá: formação de monitores*” (Health literacy among students aged 15 to 18 in sexual and reproductive health in the public school system in the state of Amapá: training of monitors). The project aimed to analyze adolescents’ HL level regarding sexual and reproductive health in the context of teen pregnancy and the occurrence of STIs to support the training of monitors in the same social group. Based on the results of this research, it was identified the need to create an educational booklet to facilitate adolescents’ understanding of the topic.

To assess adolescent students’ HL level, an instrument originating from the S-TOFHLA was applied, already adapted and used to analyze adolescents’ HL level^(10,11)^, also by the authors for this study, with the purpose of assessing the ways in which adolescents perceive, understand, and use information about sexual and reproductive health.

The questionnaire consisted of six closed-ended questions about STIs (1 - types of STIs; 2 - ways to prevent STIs; 3 - methods used to prevent pregnancy; 3 - ways STIs are transmitted; 4 - adolescents’ general knowledge about STI transmission; 5 - adolescents’ knowledge about types of relationships; 6 - adolescents’ knowledge about risk behavior). A correct answer corresponds to a score of one, and a wrong answer, a score of zero. HL is considered inadequate when the score ranges from zero to two, average, from three to four, and adequate, from five to six correct answers.

The second stage consisted of a literature review, which aimed to identify scientific evidence on STIs and risk factors. The search was conducted in the Medical Literature Analysis and Retrieval System online (via PubMed^®^), Scopus, Latin American Literature on Caribbean and Health Sciences, Bibliographic Index of Health Sciences, and the Nursing Database via the Virtual Health Library electronic databases^(12)^. The acronym PICo (Population, Phenomenon of Interest, Context) was used to develop the research question. The PICo strategy elements consist of: P - adolescents; I - STIs; Co - reproductive health/sexual behavior. Thus, the guiding question of the study was: what are the main STIs and their impact on adolescents’ reproductive health?

Full-text studies, from primary sources, without language restrictions, that presented aspects related to the prevalence of STIs in adolescents and their relationship with sexual behaviors and health repercussions, were included in the five-year period (2018-2023). This period was justified by the search for more recent publications. Editorials, theses, dissertations, review articles, and those not addressing the research topic were excluded. Identifying the prevalence of STIs in adolescents allows us to identify associated risky sexual behaviors and delve deeper into contexts of vulnerability, thus prioritizing information and contextualizing health education strategies.

The keywords and descriptors used for the search were “Adolescent”, “Adolescents”, “Adolescence”, “Prevalence” and “Sexual Behavior”. They were consulted in the Health Sciences Descriptors and Medical Subject Headings.

After completing the integrative review, the studies found were refined to define the main topics to be addressed in the booklet, associating them with the data obtained from the S-TOFHLA. Based on this analysis and the exploration of methodologies and topics used in the Ministry of Health manuals, relevant topics were identified that needed to be better addressed with the booklet’s target audience.

The booklet was created according to the recommendations for the development and effectiveness of educational technologies, considering, during the process of creating the material, organization, content, clear and succinct language, paying attention to the target audience’s reality in relation to the educational level, in order to create a material with understandable information, with simple and easy-to-understand language^(13)^.

To create the booklet’s visual aspect, the researchers created illustrations using Procreate - Sketch, Paint, Create, and Clip Studio Paint, all programs specifically designed for graphic design.

The final version of the booklet is 34 pages long, including a cover, back cover, table of contents, introductions, and the content itself. The booklet was made available online and in print, with the print version using 115-gram matte coated paper and both front and back pages.

### Analysis of results and statistics

Data relating to the first stage of the research were compiled into electronic spreadsheets in Microsoft Excel 2010 and, after coding and tabulation, analyzed using descriptive statistics in the Statistical Package for the Social Sciences version 29.0.1.0 of 2023. Descriptive statistical analysis revealed the absolute and relative frequencies for each variable.

## RESULTS

### Assessment of school adolescents’ literacy level

As for assessment of adolescent students’ literacy level, it was identified that only 49.3% (n=275) of adolescents demonstrated knowledge about what STIs are, and 47.3% (n=264) were unable to correctly identify, given the S-TOFHLA options, which were or were not STIs. When assessed on what adolescents understood about forms of prevention, 47.3% of adolescents (n=264) marked the alternative “morning-after pill” as incorrect. The others marked the options “not having multiple sexual partners” (20.8%; n=116), “not sharing syringes” (17.1%; n=95), “use of the female condom” (11.6%; n=65) and did not answer the question (3.2%; n=18).

Considering the forms of STI transmission, 53.4% (n=298) of participants marked the alternative “using public drinking fountains”, 19.7% (n=110), “sexual practice without a condom”, 13.6% (n=76), “vertical transmission”, and 9.7% (n=54), “sharing syringes”. For this question, it was found that 3.6% (n=20) did not mark any option.

Concerning adolescents’ general knowledge about STIs, 66.1% (n=369) consider condom use to be the best way to prevent them, while 18.3% (n=102) consider birth control pills; 9.1% (n=51) believe there is no risk of transmission during breastfeeding; 4.1% (n=23) consider there is no risk of STI transmission among adolescents; and 2.3% (n=13) did not answer.

In relation to relationship types, 42.7% of adolescents (n=238) stated that maintaining a faithful relationship helps reduce exposure to STIs, but 11.5% (n=64) stated that having multiple sexual partners does not contribute to reducing these. Furthermore, 19% of adolescents (n=238) considered contraceptives a form of prevention; 12.2% (n=68) did not consider syphilis, chlamydia, gonorrhea, and HPV to be STIs; and 14.7% (n=82) did not know how to answer.

Regarding risk behaviors, 37.1% (n=207) recognized the importance of using condoms even during the first sexual relations; 27.1% (n=151) believed that maintaining a relationship with a single partner reduces the risk of infection; 11.8% (n=66) believed that taking a shower after sexual intercourse reduces the chances of pregnancy and STIs; 7.7% considered that only oral sex will prevent STIs; and 16.3% (n=91) did not know how to answer.

Based on these results, it was found that only 8.1% of adolescents (n=45) have an adequate HL level, 36.2% (n=202) have an average level, and 55.7% (n=311) have an inadequate level, as shown in Table 1.

**Table 1:** Score of school adolescents in relation to health literacy level

Score	n	%
Adequate HL Average HL Inadequate HL Total	45 202 311 558	8.1 36.2 55.7 100

*HL - health literacy.*

### Bibliographic survey

The literature survey identified a total of 36,096 studies, and after filtering, 4,753 remained for reading their titles and abstracts, with 82 articles selected for full text. After applying the inclusion and exclusion criteria, 36 articles were included in the sample. After reading the articles, the results were categorized into two categories: a) STI prevalence; and b) Relationship between risk behaviors and STIs.

In the first strand, syphilis, HIV, viral hepatitis, and vulvovaginal infections such as chlamydia, candidiasis, gonorrhea, and trichomoniasis stood out as the main STIs affecting adolescents. Meanwhile, in the second strand, it was also identified that risky sexual behaviors among adolescents, such as early initiation of sexual activity, multiple sexual partners, lack of or inadequate use of condoms, drug use, low education and socioeconomic status, lack of information, and lack of access to health services, have led to increased STI rates^(10)^.

### Educational booklet development

**Figure 1:**
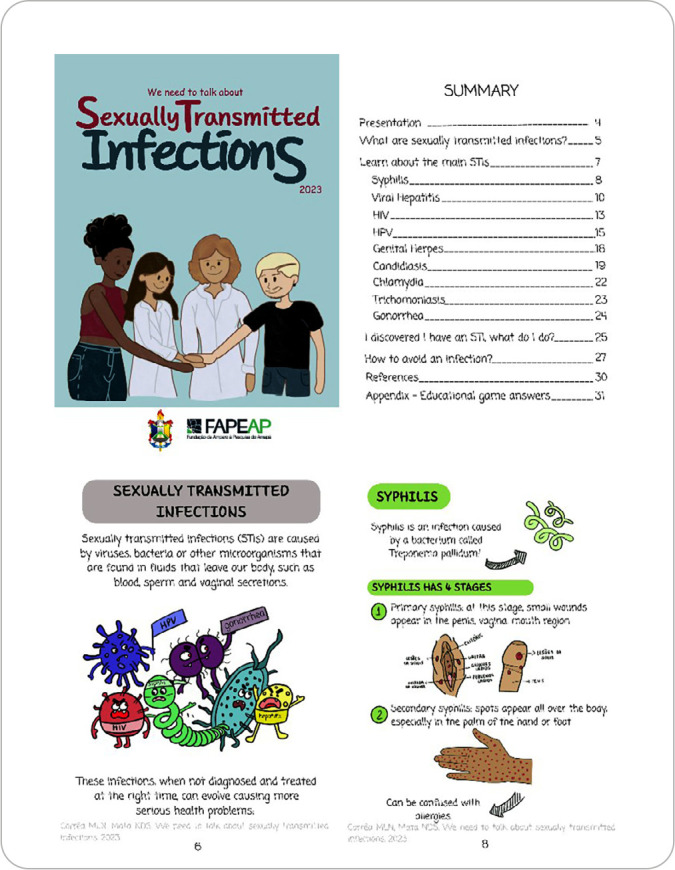
Cover and some pages of the booklet entitled “We need to talk about sexually transmitted infections”

Based on the above data, an educational booklet entitled “*Precisamos falar sobre Infecções Sexualmente Transmissíveis*” (We need to talk about sexually transmitted infections) was developed. It is 34 pages long and measures 210 x 148 x 5 mm (A5 size). The cover features the title and a picture of four characters: a teacher, a nursing student, and an adolescent couple, all holding hands, representing the partnership between healthcare professionals and adolescents in STI prevention (Figure 1).

On the back page of the booklet, the cataloging card contains the coat of arms of the higher education institution, the funding institution, the authors’ names, and technical credits. After the presentation page, there is the summary, which shows that the booklet’s content has been divided into four sections, intended to present a logical sequence to the target audience. These sections are, respectively: 1) What are STIs?; 2) Learn about the main STIs; 3) I discovered I have an STI, what should I do?; 4) How can I prevent a new infection?

**Figure 2:**
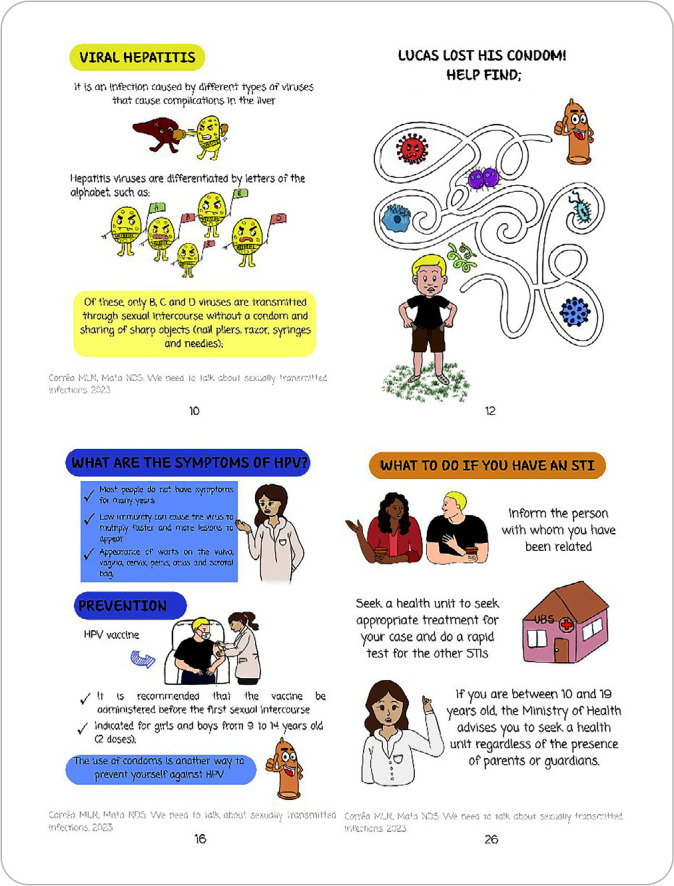
Some pages from the booklet entitled “We need to talk about sexually transmitted infections”

The first topic of the booklet, “What are STIs?”, provided a brief general explanation of what causes STIs and what they can cause. The next topic, addressing the main STIs, covered syphilis, viral hepatitis, HIV, HPV, genital herpes, candidiasis, chlamydia, trichomoniasis, and gonorrhea. For each of these, we discussed their meaning, main symptoms, and how to identify and prevent them. For every two sessions addressing STIs, a session with educational games was developed, helping to reinforce the content (Figure 2).

In the third topic, “I discovered I had an STI, what should I do?”, some health guidelines are provided, such as advising the person who had sexual intercourse to seek a health facility for diagnosis and appropriate treatment, and instructing them to seek a Basic Health Unit regardless of the presence of their parents and/or guardians. However, it is important to emphasize that, even though the Ministry of Health and the Child and Adolescent Statute recommend that healthcare professionals provide care for unaccompanied adolescents, aiming at their own self-care, each case must be assessed individually and the presence of parents and/or guardians requested, when applicable^(14)^.

In the topic about prevention, “How do I avoid a new infection?”, male and female condoms were discussed, and it was decided to include images containing step-by-step instructions on their proper use and correct disposal.

The images in the booklet were produced by the researchers themselves, who sought to combine informative content with clarity and objectivity, as very long materials can become tiresome, and with language accessible to the target audience. Bibliographic references were included at the end. All pages were sequentially numbered, with the booklet reference added to the bottom margin.

## DISCUSSION

The use of educational booklets has been identified as a teaching strategy that fosters the development of health knowledge, providing a suitable tool for raising awareness and promoting individual responsibility, using accessible language and visual aids that facilitate understanding. Studies conducted with adolescents found that they possessed information about STIs; however, this information was superficial, inaccurate, and obtained from unreliable sources^(15)^.

Thus, the literature review was important for the booklet text elaboration, as it was possible to identify the main risk behaviors that contribute to the increase in STIs, the main ones being multiple partners, non-use of condoms, non-performance of rapid tests, use of illicit or licit drugs, and early first sexual intercourse^(16,17)^.

Furthermore, a key factor in the success of booklets is their adaptation to the target audience’s literacy level, since a moderate or inadequate literacy level means less understanding of the importance of preventive measures and difficulty adopting healthy lifestyle habits, unlike an individual with a satisfactory literacy level. Many adolescents have limited reading and comprehension skills, which can compromise their understanding of the messages conveyed^(18)^.

It was also observed that most adolescents had an inadequate level of literacy regarding ways to prevent and transmit STIs, particularly unaware of the possibility of transmission through other means. This lack of information can be attributed to the lack of in-depth education on the subject with these adolescents in schools or even at home, which also contributes to increased vulnerability^(19)^.

However, educational booklets have the ability to bring scientific evidence closer to the lay public through various strategies to the point that users/readers, even those with low levels of education or even reading difficulties, are able to understand what is contained in the educational material. They are also considered strategic tools for promoting prevention, expanding the reach of reliable information and demystifying taboos that often hinder dialogue about sexual health^(20)^.

Printed or digital educational materials are presented as devices that contribute to the communication process in health education practices. However, the advantage of printed resources is their ability to promote health, as they are a tangible tool, in which all the information contained can be easily viewed, in order to improve content assimilation, when compared to isolated verbal guidelines^(6,15)^.

Another positive aspect of using educational booklets is their versatility, as they can be used in a variety of settings and by different professionals, such as teachers and healthcare professionals, to disseminate information. Activities such as lectures, workshops, and discussion groups complement the written material, allowing for a more interactive and dynamic approach^(6)^.

### Study limitations

The limitation of this study highlights the lack of booklet validity by expert judges in the area and by adolescents, which will be carried out in a future study.

### Contributions to nursing, health or public policy

The study contributes significantly to driving changes in the care provided by nursing professionals, as the use of educational materials, such as educational booklets, ensures the development of preventive practices and management of STIs. It is noteworthy that developing material based on scientific evidence facilitates the health education process and enables its use in public health services.

## CONCLUSIONS

The results showed that most of the adolescents assessed presented an inadequate HL level related to sexual and reproductive health, especially regarding knowledge about STIs. This finding may be directly associated with the adoption of risk behaviors and the high incidence of these infections among adolescents, constituting a public health problem that requires targeted and contextualized educational interventions.

By identifying the knowledge gaps demonstrated by incorrect responses to the questionnaire, it was possible to design and develop an educational and structured booklet based on up-to-date scientific evidence, accessible language, and culturally appropriate for the target audience. The material aims not only to address the identified informational deficiencies but also to act as a facilitating tool for the development of critical thinking and autonomy among adolescents in decisions related to their sexual and reproductive health.

In addition to its informative nature, the booklet represents a low-cost, easily replicable, and accessible educational technology, available in print and digital formats, with the potential to be incorporated into pedagogical practices and health promotion strategies in school settings. Therefore, this study contributes both to the situational diagnosis of adolescents’ HL and to the proposal of concrete and applicable educational solutions. Future research is recommended to assess the booklet’s effectiveness in different contexts and explore its integration into institutional health education programs, strengthening the intersectoral collaboration between health and education.

## Data Availability

The research data are available within the article.
