# Supplementary material for: Development of educational technology on sexually transmitted infections for adolescent students
Source: Rev Bras Enferm. 2026 Mar 16;79:e20250024. doi: 10.1590/0034-7167-2025-0024 (PMC12995331; doi:10.1590/0034-7167-2025-0024)
Supplement: Translation

# Translation: Desenvolvimento de tecnologia educativa sobre infecções sexualmente transmissíveis para adolescentes escolares

**DOI:** 10.1590/0034-7167-2025-0024pt

**Authors:** Mayra Loreanne Nascimento Corrêa, Maria Eduarda dos Santos Alves, Pablo Palmerim Santana, Vinícius dos Santos Maciel, Angélica Gonçalves Silva Belasco, Tatiana do Socorro dos Santos Calandrini, Camila Rodrigues Barbosa Nemer, Nely Dayse Santos da Mata

**Affiliations:** I Universidade Federal do Amapá. Macapá, Amapá, Brasil; II Universidade Federal de São Paulo. São Paulo, São Paulo, Brasil

**Keywords:** Adolescente, Infecções Sexualmente Transmissíveis, Letramento em Saúde, Tecnologia Educacional, Educação em Saúde.

## Abstract

**Objetivos:**

descrever a construção de cartilha educativa para adolescentes escolares sobre as principais infecções sexualmente transmissíveis.

**Métodos:**

pesquisa metodológica, com abordagem quantitativa, realizada em quatro etapas: 1. Avaliação do nível de letramento em saúde dos adolescentes escolares com base no instrumento *Short Test of Functional Health Literacy in Adults*; 2. Levantamento bibliográfico; 3. Análise de conteúdo e elaboração textual; 4. Construção da cartilha. A pesquisa seguiu o *checklist STrengthening the Reporting of OBservational studies in Epidemiology*.

**Resultados:**

apenas 8,1% dos adolescentes possuem nível de letramento em saúde adequado. A cartilha foi dividida em: o que são infecções sexualmente transmissíveis?; Conheça as principais infecções sexualmente transmissíveis; Descobri que estou com uma infecção sexualmente transmissível, o que eu faço?; Como faço para evitar uma nova infecção?

**Conclusões:**

a cartilha sobre infecções sexualmente transmissíveis mostra potencial para contribuir com as práticas de educação em saúde, facilitando o entendimento da temática pelos adolescentes.

## INTRODUÇÃO

O Ministério da Saúde considera a definição utilizada pela Organização Mundial da Saúde, que caracteriza a adolescência como o período de 10 a 19 anos, e compreende como juventude a população da faixa etária de 15 a 24 anos^(1)^. Esse período é marcado por diversas mudanças biológicas, psicológicas e sociais intensas, em que ocorre o início da vida sexual para muitos jovens. Estudos epidemiológicos indicam que adolescentes representam um grupo vulnerável e de risco para contrair infecções sexualmente transmissíveis (ISTs) devido a diversos fatores, como o início precoce da vida sexual, múltiplos parceiros sexuais e uso irregular de preservativos^(2,3)^.

Associado a isso, muitos adolescentes não possuem um entendimento adequado sobre os riscos e as formas de prevenção das ISTs, o que contribui diretamente para a disseminação dessas infecções, aumentando a vulnerabilidade desse grupo. Essa falta de compreensão é agravada pelo estigma e tabu que ainda cercam o tema da sexualidade na sociedade. Muitas vezes, adolescentes não encontram um ambiente seguro para discutir suas dúvidas e preocupações, seja com familiares, amigos ou mesmo no contexto escolar. Essa barreira comunicacional faz com que muitos jovens busquem informações em fontes não confiáveis ou, em alguns casos, nem sequer busquem informações, adotando práticas sem o devido conhecimento^(4)^.

Essa falta de entendimento também pode ser compreendida como a ausência de letramento em saúde (LS), que se refere à capacidade dos indivíduos de entender, avaliar e utilizar informações de saúde para tomar decisões a respeito de sua saúde. No contexto das ISTs, pode abranger o conhecimento sobre como estas são transmitidas, os métodos de prevenção, e o reconhecimento de sinais e sintomas. Quando o indivíduo tem acesso a informações claras e adaptadas à sua realidade, ele se sente mais empoderado e capaz de adotar medidas preventivas, buscar ajuda profissional quando necessário e desmistificar estigmas que podem atrasar o diagnóstico e tratamento^(5)^.

Assim, entende-se que a falta de acesso à informação em saúde sobre as ISTs é uma questão-chave que tem contribuído para a alta prevalência dessas infecções em adolescentes. Dessa forma, há mais probabilidade de um indivíduo com adequado LS ter melhores condições de saúde quando comparado àquele com inadequado LS, que possui mais dificuldade em compreender as orientações a respeito de medidas preventivas e a própria saúde^(5)^.

Diante disso, enfatiza-se a necessidade de desenvolver estratégias educativas que promovam o entendimento e a conscientização sobre ISTs em adolescentes. As tecnologias educativas, como *folders* e cartilhas, quando direcionadas ao adolescente, são recursos que possibilitam potencializar o aprendizado de forma lúdica, promovendo uma mudança de comportamento que fortaleça a proteção da saúde sexual dos jovens e contribua para a redução das taxas de infecção por IST nessa faixa etária^(6,7)^.

## OBJETIVOS

Descrever a construção de cartilha educativa para adolescentes escolares sobre as principais ISTs.

## MÉTODOS

### Aspectos éticos

A pesquisa seguiu as normas da Resolução n^o^ 466/12 do Conselho Nacional de Saúde, que estabelece diretrizes e normas regulamentadoras quanto aos aspectos éticos da pesquisa, e foi submetida e aprovada por Comitê de Ética em Pesquisa. Todos os participantes da pesquisa assinaram o Termo de Consentimento Livre e Esclarecido e o Termo de Assentimento Livre e Esclarecido, no qual foram assinadas duas vias (originais). Uma via ficou com os participantes, e a outra, com os pesquisadores.

### Desenho, período e local do estudo

Trata-se de pesquisa metodológica de abordagem quantitativa desenvolvida em quatro etapas^(8,9)^: 1. Avaliação do nível de LS dos adolescentes escolares com base no instrumento *Short Test of Functional Health Literacy in Adults* (S-TOFHLA); 2. Levantamento bibliográfico; 3. Análise de conteúdo e elaboração textual; 4. Construção da cartilha. A pesquisa seguiu o *checklist STrengthening the Reporting of OBservational studies in Epidemiology*.

Este estudo foi desenvolvido entre agosto de 2022 e maio de 2023 em 11 escolas públicas localizadas em Macapá, capital do estado do Amapá, divididas em escolas estaduais e municipais.

### População ou amostra; critérios de inclusão e exclusão

Participaram do estudo 558 adolescentes escolares de 11 escolas públicas no município de Macapá. Os participantes foram selecionados por conveniência durante o período de coleta de dados, por meio de convite feito pessoalmente para participar da pesquisa pelos pesquisadores nas salas de aula, explicando detalhadamente e claramente sobre o que era a pesquisa e seus objetivos.

Em cada uma das escolas, havia cerca de 350 alunos dentro da faixa etária estabelecida, resultando em um total aproximado de 3.850 adolescentes. A escolha das escolas ocorreu com base na indicação da Secretaria de Educação, sendo que, para a viabilidade do projeto, foram selecionadas duas escolas por zona geográfica (norte, sul, leste e oeste), resultando em seis são escolas estaduais e cinco, escolas municipais.

O cálculo amostral não foi realizado previamente, considerando a natureza exploratória e a viabilidade da pesquisa em parceria com as escolas. No entanto, considerando uma população estimada de 3.850 adolescentes matriculados nas 11 escolas participantes, a amostra final de 558 estudantes representa aproximadamente 14,5% da população elegível, o que confere boa representatividade para o diagnóstico local.

Adotaram-se como critérios de inclusão ter entre 15 e 18 anos, estar matriculado em escola pública estadual ou municipal do estado do Amapá e frequentar regularmente as aulas. Estabeleceram-se como critérios de exclusão adolescentes com comprometimento cognitivo autodeclarado, informado pela escola, ou que mudaram de escola durante o período da pesquisa.

### Protocolo do estudo

A primeira etapa é integrada ao projeto de pesquisa intitulado “Letramento em saúde entre escolares de 15 a 18 anos na saúde sexual e reprodutiva da rede pública no estado do Amapá: formação de monitores”, o qual teve como objetivo analisar o nível de LS de adolescentes acerca de saúde sexual e reprodutiva no contexto de gravidez precoce e ocorrência de ISTs para subsidiar a formação de monitores no mesmo grupo social. A partir dos resultados desta pesquisa, verificou-se a necessidade da criação de uma cartilha educativa para facilitar o entendimento da temática pelos adolescentes.

Para avaliação desse nível de LS dos adolescentes escolares, aplicou-se instrumento com origem no S-TOFHLA, já adaptado e utilizado para analisar o nível de LS de adolescentes^(10,11)^, também pelos autores para este estudo, com a finalidade de avaliar as formas como o adolescente percebe, compreende e utiliza a informação sobre a saúde sexual e reprodutiva.

O questionário se compôs de seis perguntas fechadas sobre as ISTs (1 - sobre os tipos de ISTs; 2 - sobre as formas de prevenção das ISTs; 3 - métodos utilizados para evitar a gravidez; 3 - sobre as formas de transmissão das ISTs; 4 - conhecimento geral dos adolescentes sobre a transmissão das ISTs; 5 - sobre o conhecimento dos adolescentes quanto às formas de relacionamentos; 6 - conhecimento dos adolescentes quanto ao comportamento de risco). A resposta certa corresponde à pontuação um, e a errada, à pontuação zero. O LS é considerado inadequado quando a pontuação vai de zero a dois, mediano, de três a quatro, e adequado, de cinco a seis acertos.

A segunda etapa consistiu no levantamento bibliográfico, que teve como objetivo identificar as evidências científicas sobre ISTs e os fatores de risco. A busca foi realizada nas bases eletrônicas de dados *Medical Literature Analysis and Retrieval System online* (via PubMed^®^), Scopus, Literatura Latino-Americana de Ciências do Caribe e da Saúde, Índice Bibliográfico de Ciências da Saúde e Banco de Dados em Enfermagem via Biblioteca Virtual em Saúde^(12)^. Para elaboração da questão de pesquisa, empregou-se o acrônimo PICo (População, Fenômeno de Interesse, Contexto). Os elementos da estratégia PICo consistem em: P - adolescentes; I - ISTs; Co - saúde reprodutiva/comportamento sexual. Dessa forma, a pergunta norteadora do estudo foi: quais as principais ISTs e suas repercussões para a saúde reprodutiva de adolescentes?

Foram incluídos estudos completos, de fontes primárias, sem restrições de idioma, que apresentassem aspectos relacionados à prevalência de ISTs em adolescentes e sua relação com comportamentos sexuais e repercussões para sua saúde, no período de cinco anos (2018-2023). O período é justificado pela busca de publicações mais atuais. Foram excluídos os registros editoriais, teses, dissertações, artigos de revisão e os que não contemplam o objeto pesquisado. A identificação da prevalência de ISTs em adolescentes permite identificar comportamentos sexuais de risco associados e aprofundar contextos de vulnerabilidade, priorizando, assim, informações e contextualizando estratégias de educação em saúde.

As palavras-chave e descritores utilizados para busca foram “Adolescente” (*Adolescent*), “Adolescentes” (*Adolescents*), “Adolescência” (*Adolescence*), “Prevalência” (*Prevalence*) e “Comportamento Sexual” (*Sexual Behavior*). Estes foram consultados nos Descritores em Ciências da Saúde e *Medical Subject Headings*.

Após a finalização da revisão integrativa, realizou-se um refinamento dos estudos encontrados para definir os principais assuntos a serem abordados na cartilha, associando-os com os dados obtidos a partir da aplicação do S-TOFHLA. Com base nessa análise e na exploração das metodologias e temas utilizados nos manuais do Ministério da Saúde, identificaram-se os assuntos pertinentes que precisavam ser mais bem abordados com o público-alvo na cartilha.

A cartilha foi confeccionada conforme as recomendações para elaboração e eficácia de tecnologias educativas, considerando-se, durante o processo de construção do material, organização, conteúdo, linguagem clara e sucinta, atentando para a realidade do público-alvo em relação ao nível educacional, a fim de se construir um material com informações compreensíveis, com linguagem simples e de fácil entendimento^(13)^.

Para compor a parte visual da cartilha, as ilustrações foram confeccionadas pelos pesquisadores. Foram utilizados os programas *Procreate - Sketch, Paint, Create* e *Clip Studio Paint,* desenvolvidos especificamente para a criação de *designs* gráficos.

A versão final da cartilha possui 34 páginas, contendo capa, contracapa, sumário, apresentações e o conteúdo propriamente dito. Optou-se por disponibilizar a cartilha em versão *online* e impressa, sendo que, na versão impressa, escolheu-se usar o papel couchê fosco de 115 gramas e utilizar frente e verso da folha.

### Análise dos resultados e estatística

Os dados referentes à primeira etapa da pesquisa foram compilados em planilhas eletrônicas no *Microsoft Excel* 2010 e, após codificação e tabulação, analisados por meio de estatística descritiva no *software Statistical Package for the Social Sciences*, versão 29.0.1.0 de 2023. A análise estatística descritiva revelou as frequências absolutas e relativas para cada variável.

## RESULTADOS

### Avaliação do nível de letramento dos adolescentes escolares

No que se refere à avaliação do nível de letramento dos adolescentes escolares, identificou-se que apenas 49,3% (n=275) dos adolescentes demonstraram ter conhecimento sobre quais são as ISTs, e 47,3% (n=264) não souberam identificar corretamente, diante das opções do S-TOFHLA, quais eram ou não ISTs. Quando avaliados sobre o que os adolescentes entendiam sobre formas de prevenção, 47,3% dos adolescentes (n=264) marcaram como incorreta a alternativa “pílula do dia seguinte”. Os demais marcaram as opções “não ter vários parceiros sexuais” (20,8%; n=116), “não compartilhar seringas” (17,1%; n=95), “uso do preservativo feminino” (11,6%; n=65) e não responderam o questionamento (3,2%; n=18).

Considerando as formas de transmissão das ISTs, 53,4% (n=298) dos participantes marcaram a alternativa “utilizar bebedouros públicos”, 19,7% (n=110), “prática sexual sem preservativo”, 13,6% (n=76), “transmissão vertical”, e 9,7% (n=54), “compartilhamento de seringa”. Para esse questionamento, obteve-se que 3,6% (n=20) não assinalaram nenhuma opção.

Em relação ao conhecimento geral dos adolescentes sobre as ISTs, 66,1% (n=369) consideram o uso de preservativo a melhor forma de se prevenir, enquanto 18,3% (n=102) consideram as pílulas anticoncepcionais; 9,1% (n=51) acreditam que não haja riscos de transmissão durante a amamentação; 4,1% (n=23) consideram que não há risco de contágio de IST entre adolescentes; e 2,3% (n=13) não responderam.

No que tange às formas de relacionamento, 42,7% dos adolescentes (n=238) alegaram que manter um relacionamento fiel ajuda a reduzir a exposição às ISTs, porém 11,5% (n=64) afirmaram que ter vários parceiros sexuais não contribui para a redução destas. Além disso, 19% dos adolescentes (n=238) consideravam o anticoncepcional uma forma de se prevenir; 12,2% (n=68) não consideravam sífilis, clamídia, gonorreia e HPV como IST; e 14,7% (n=82) não souberam responder.

Quanto aos comportamentos de risco, 37,1% (n=207) reconheciam a importância do uso do preservativo mesmo durante as primeiras relações sexuais; 27,1% (n=151) acreditavam que manter relacionamento com parceiro único diminui o risco de infecção; 11,8% (n=66) acreditam que tomar banho após a relação sexual diminui as chances de gravidez e IST; 7,7% consideravam que somente o sexo oral irá prevenir contra IST; e 16,3% (n=91) não souberam responder.

Com base nesses resultados, verificou-se que apenas 8,1% dos adolescentes (n=45) possuem nível de LS adequado, 36,2% (n=202), mediano, e 55,7% (n=311), inadequado, conforme apresentado na Tabela 1.

**Tabela 1:** Pontuação dos adolescentes escolares em relação ao nível de letramento em saúde

Pontuação	n	%
LS adequado LS mediano LS inadequado Total	45 202 311 558	8,1 36,2 55,7 100

*LS - letramento em saúde.*

### Levantamento bibliográfico

No levantamento bibliográfico, identificou-se no total 36.096 estudos e, após a aplicação dos filtros, restaram 4.753 para leitura dos títulos e resumos, sendo selecionados 82 artigos para leitura na íntegra. Após aplicação dos critérios de inclusão e exclusão, 36 artigos foram incluídos na amostra. Após a leitura dos artigos, os resultados foram categorizados em duas vertentes: a) Prevalência das ISTs; e b) Relação entre os comportamentos de riscos e as ISTs.

Na primeira vertente, destacaram-se a sífilis, HIV, hepatites virais e infecções vulvovaginais, como a clamídia, candidíase, gonorreia e tricomoníase como as principais ISTs que afetam os adolescentes. Enquanto isso, na segunda vertente, identificou-se ainda que os comportamentos sexuais de risco entre adolescentes, como o início precoce da atividade sexual, múltiplos parceiros sexuais, falta de uso ou uso inadequado de preservativos, uso de drogas, baixa escolaridade e nível socioeconômico, falta de informação, e acesso aos serviços de saúde, têm levado ao aumento das taxas de ISTs^(10)^.

### Construção da cartilha educativa

Com base nos dados acima, foi elaborada a cartilha educativa intitulada “Precisamos falar sobre Infecções Sexualmente Transmissíveis”, expressa em 34 páginas, com dimensões de 210x148 e 5 mm (tamanho A5). A capa contém o título e uma figura com quatro personagens, sendo eles uma professora, uma acadêmica de enfermagem e um casal de adolescentes, todos de mãos dadas, representando a união entre profissionais de saúde e adolescentes na prevenção das ISTs (Figura 1).

**Figura 1:** Capa e algumas páginas da cartilha intitulada “Precisamos falar sobre Infecções Sexualmente Transmissíveis”

Na página posterior da cartilha, encontra-se a ficha catalográfica, com a inserção do brasão da instituição de ensino superior, da instituição financiadora, o nome dos autores e créditos técnicos. Após a página de apresentação, tem-se o sumário, no qual é possível observar que o conteúdo da cartilha foi delimitado em quatro tópicos, com o intuito de apresentar uma sequência lógica ao público-alvo. São eles, respectivamente: 1) O que são ISTs?; 2) Conheça as principais ISTs; 3) Descobri que estou com uma IST, o que eu faço?; 4) Como faço para evitar uma nova infecção?.

No primeiro tópico da cartilha, correspondente ao “O que são ISTs?”, foi apresentada uma breve explicação geral sobre o que causam as ISTs e o que podem causar. No tópico seguinte, referente às principais ISTs, foram abordados sífilis, hepatites virais, HIV, HPV, herpes genital, candidíase, clamídia, tricomoníase e gonorreia. Para cada um deles, discutimos o que são, os principais sintomas e como identificá-los e preveni-los. A cada duas sessões abordando as ISTs, foi elaborada uma sessão com jogos educativos, contribuindo para a fixação do conteúdo (Figura 2).

**Figura 2:** Algumas páginas da cartilha intitulada “Precisamos falar sobre Infecções Sexualmente Transmissíveis”

No terceiro tópico, “Descobri que estou com uma IST, o que eu faço?”, são dadas algumas orientações de saúde, como orientar a pessoa que manteve relações sexuais que procure uma unidade para realizar o diagnóstico e tratamento adequado, sendo instruída a procurar uma Unidade Básica de Saúde independente da presença dos pais e/ou responsáveis. No entanto, é importante ressaltar que, mesmo que o Ministério da Saúde e o Estatuto da Criança e do Adolescente orientem o atendimento pelos profissionais de saúde de adolescentes desacompanhados, visando ao protagonismo de seu próprio cuidado, deve-se avaliar cada caso individualmente e solicitar a presença dos pais e/ou responsáveis, quando pertinente^(14)^.

No tópico acerca da prevenção, “Como faço para evitar uma nova infecção?”, foram discutidos os preservativos masculino e feminino e optou-se por inserir imagens contendo o passo a passo da utilização adequada e quanto ao descarte correto dos mesmos.

As imagens contidas na cartilha foram produzidas pelos próprios pesquisadores, que buscaram aliar conteúdo informativo com clareza e objetividade, visto que materiais muito extensos se tornam cansativos e com linguagem acessível ao público-alvo. Ao final, foram inseridas as referências bibliográficas. Todas as páginas foram sequencialmente numeradas, acrescentando-se a referência da cartilha na margem inferior delas.

## DISCUSSÃO

A utilização de cartilhas educativas tem sido identificada como uma estratégia de ensino que favorece o desenvolvimento do conhecimento em saúde, por meio de um instrumento propício para sensibilizar o leitor e promover a responsabilidade individual, utilizando uma linguagem acessível e recursos visuais que facilitam o entendimento. Nos estudos realizados com adolescentes, constatou-se que esses possuem informações sobre ISTs; no entanto, essas informações eram superficiais, imprecisas e obtidas por fontes pouco confiáveis^(15)^.

Dessa forma, o levantamento da literatura foi importante para a elaboração textual da cartilha, pois foi possível identificar os principais comportamentos de riscos que contribuem para o aumento das ISTs, sendo os principais multiplicidade de parceiros, não utilização de preservativo, não realização de testes rápidos, utilização de drogas ilícitas ou lícitas e precocidade da primeira relação sexual^(16,17)^.

Além disso, um ponto importante para o sucesso das cartilhas é sua adaptação ao nível de letramento do público-alvo, uma vez que o nível de letramento mediano ou inadequado significa menor entendimento da importância de medidas preventivas e dificuldade na adoção de hábitos de vida saudáveis, diferentemente de um indivíduo com nível de letramento satisfatório. Muitos adolescentes possuem habilidades limitadas de leitura e interpretação, o que pode comprometer a compreensão das mensagens transmitidas^(18)^.

Observou-se ainda que a maioria dos adolescentes apresentou um nível de letramento inadequado acerca das formas de prevenção e transmissão das ISTs, desconhecendo, principalmente, a possibilidade do contágio por outros meios. Essa ausência de informações pode ser atribuída à falta de aprofundamento sobre o assunto com esses adolescentes nas escolas ou até mesmo no espaço familiar, que também contribui para o aumento da vulnerabilidade^(19)^.

No entanto, as cartilhas educativas possuem capacidade de aproximar evidências científicas do público leigo, por meio de diversas estratégias, ao ponto de o usuário/leitor, mesmo tendo baixa escolaridade ou até mesmo dificuldade de leitura, seja capaz de compreender o que consta no material educativo. Também são consideradas ferramentas estratégicas para promover a prevenção, ampliando o alcance de informações confiáveis e desmistificando tabus que muitas vezes dificultam o diálogo sobre saúde sexual^(20)^.

Materiais educativos impressos ou digitais se apresentam como dispositivos que contribuem no processo de comunicação em práticas de educação em saúde, entretanto a vantagem dos recursos impressos é sua capacidade de promover a saúde, pois são uma ferramenta palpável, na qual todas as informações contidas podem ser facilmente visualizadas, de forma a melhorar a assimilação do conteúdo, quando comparados a orientações verbais isoladas^(6,15)^.

Destaca-se, ainda, como ponto positivo da utilização das cartilhas educativas a versatilidade das mesmas, uma vez que podem ser utilizadas em diversos ambientes e por diferentes profissionais, como professores e profissionais de saúde, que visem à propagação de informações. Atividades como palestras, oficinas e rodas de conversa complementam o material escrito, permitindo uma abordagem mais interativa e dinâmica^(6)^.

### Limitações do estudo

A limitação do presente estudo destaca a não validação da cartilha por juízes especialistas na área e pelos adolescentes, o que será realizado em um próximo estudo.

### Contribuições para a área da enfermagem, saúde ou políticas públicas

O estudo contribui de forma significativa para impulsionar mudanças na assistência prestada por profissionais enfermeiros, pois, ao se utilizar materiais educativos, como as cartilhas educativas, garantem-se o desenvolvimento de práticas preventivas e enfrentamento das ISTs. Ressalta-se que, ao se construir um material baseado em evidências científicas, facilita o processo de educação em saúde e possibilita a sua utilização em serviços públicos de saúde.

## CONCLUSÕES

Os resultados evidenciaram que a maioria dos adolescentes avaliados apresentou um nível inadequado de LS relacionado à saúde sexual e reprodutiva, especialmente no que se refere ao conhecimento sobre ISTs. Esse achado pode estar diretamente associado à adoção de comportamentos de risco e à elevada incidência dessas infecções entre adolescentes, configurando um problema de saúde pública que requer intervenções educativas direcionadas e contextualizadas.

A partir da identificação das lacunas de conhecimento demonstradas nas respostas incorretas ao instrumento aplicado, foi possível idealizar e desenvolver uma cartilha educativa e estruturada com base em evidências científicas atualizadas, linguagem acessível e adequação cultural ao público-alvo. O material elaborado visa não apenas suprir as deficiências informacionais apontadas, mas também atuar como ferramenta facilitadora para o desenvolvimento do pensamento crítico e da autonomia dos adolescentes diante das decisões relacionadas à sua saúde sexual e reprodutiva.

Além de seu caráter informativo, a cartilha representa uma tecnologia educativa de baixo custo, de fácil replicação e acessibilidade, disponível em formatos impresso e digital, com potencial de ser incorporada às práticas pedagógicas e estratégias de promoção da saúde em ambientes escolares. Portanto, este estudo contribui tanto para o diagnóstico situacional do LS de adolescentes quanto para a proposição de soluções educativas concretas e aplicáveis. Recomenda-se que futuras pesquisas avaliem a efetividade da cartilha em diferentes contextos e explorem sua integração aos programas institucionais de educação em saúde, fortalecendo a atuação intersetorial entre saúde e educação.

## Data Availability

Os dados de pesquisa estão disponíveis no corpo do artigo.
